# Pathologic complete response of hepatoid adenocarcinoma of the stomach after chemo-immunotherapy: A rare case report and literature review

**DOI:** 10.3389/fsurg.2023.1133335

**Published:** 2023-03-30

**Authors:** Yunxiang Zhou, Linping Dong, Linyun Dai, Sien Hu, Yongji Sun, Yulian Wu, Tao Pan, Xiawei Li

**Affiliations:** ^1^ Department of Surgical Oncology, The Second Affiliated Hospital, Zhejiang University School of Medicine, Hangzhou, Zhejiang, China; ^2^ Key Laboratory of Cancer Prevention and Intervention, China National Ministry of Education, Cancer Institute, Second Affiliated Hospital, Zhejiang University School of Medicine, Hangzhou, Zhejiang, China; ^3^ Cancer Center, Zhejiang University, Hangzhou, Zhejiang, China; ^4^ Department of Surgery, Second Affiliated Hospital, Zhejiang University School of Medicine, Yiwu, Zhejiang, China; ^5^ Department of Surgery, Fourth Affiliated Hospital, Zhejiang University School of Medicine, Hangzhou, Zhejiang, China; ^6^ Department of Surgery, Haiyan People's Hospital, Jiaxing, Zhejiang, China

**Keywords:** hepatoid adenocarcinoma of the stomach, pathologic complete response, chemo-immunotherapy, PD-1 inhibitor, terelizumab, immune checkpoint inhibitor (ICIs)

## Abstract

**Background:**

Hepatoid adenocarcinoma of the stomach (HAS) is a highly malignant subtype of gastric carcinoma with specific clinicopathological features and extremely poor prognosis. We present an exceedingly rare case of complete response after chemo-immunotherapy.

**Case Description:**

A 48-year-old woman with highly elevated serum alpha-fetoprotein (AFP) level was found to have HAS verified by pathological examination based on gastroscopy. Computed tomography scan was done and TNM staging of the tumor was T4aN3aMx. Programmed cell death ligand-1 (PD-L1) immunohistochemistry was performed, revealing a negative PD-L1 expression. Chemo-immunotherapy including oxaliplatin plus S-1 and PD-1 inhibitor terelizumab was given to this patient for 2 months until the serum AFP level decreased from 748.5 to 12.9 ng/mL and the tumor shrank. D2 radical gastrectomy was then performed and histopathology of the resected specimen revealed that the cancerous cells had disappeared. Pathologic complete response (pCR) was achieved and no evidence of recurrence has been found after 1 year of follow-up.

**Conclusions:**

We, for the first time, reported an HAS patient with negative PD-L1 expression who achieved pCR from the combined chemotherapy and immunotherapy. Although no consensus has been reached regarding the therapy, it might provide a potential effective management strategy for HAS patient.

## Introduction

As a rare malignant neoplasm with high aggressiveness and poor prognosis, hepatoid adenocarcinoma of the stomach (HAS) accounts for 1.6%–4.3% of all gastric cancers (1) and 63% of hepatoid adenocarcinoma manifesting outside the liver (2). Due to the lack of typical early clinical symptoms, the majority of HAS patients are diagnosed at an advanced stage of the disease, with lymphatic permeation, blood vessel, and regional lymph node metastasis (3). Similar to gastric cancer, HAS is usually diagnosed histologically after endoscopic biopsy and staged using CT, endoscopic ultrasound, PET, and laparoscopy (4). Pathology is the only golden standard for diagnosing HAS, which has similar tissue morphology comparable to hepatocellular carcinoma (HCC) and frequently expresses alpha-fetoprotein (AFP) on immunohistochemistry (5–7).

Given the limited understanding of the low-incident disease, no consensus has been reached regarding the therapy of HAS so far. Although surgical resection in combination with chemotherapy is recognized as the optimal treatment for this lethal malignancy, the long-term survival remains unsatisfactory owing to the late detection, early recurrence, and aggressive biological manner of the tumor (8). In recent years, immunotherapy with immune checkpoint inhibitors (ICIs) has shown beneficial effects and good safety for various solid cancers including non-small cell lung cancer (9), gallbladder carcinosarcoma (10), bladder urothelial carcinoma (11), colorectal adenocarcinoma (12), etc. For gastric cancer, immunotherapy has also drastically improved the treatment options in the advanced phase, especially for chemorefractory gastric cancer (4, 13). However, research on immunotherapy applied to HAS has barely been reported. In this study, we, for the first time, reported an HAS patient who achieved pathologic complete response (pCR) from the combined chemotherapy and immunotherapy with negative programmed cell death ligand-1 (PD-L1) expression.

## Case presentation

**Figure 1:**
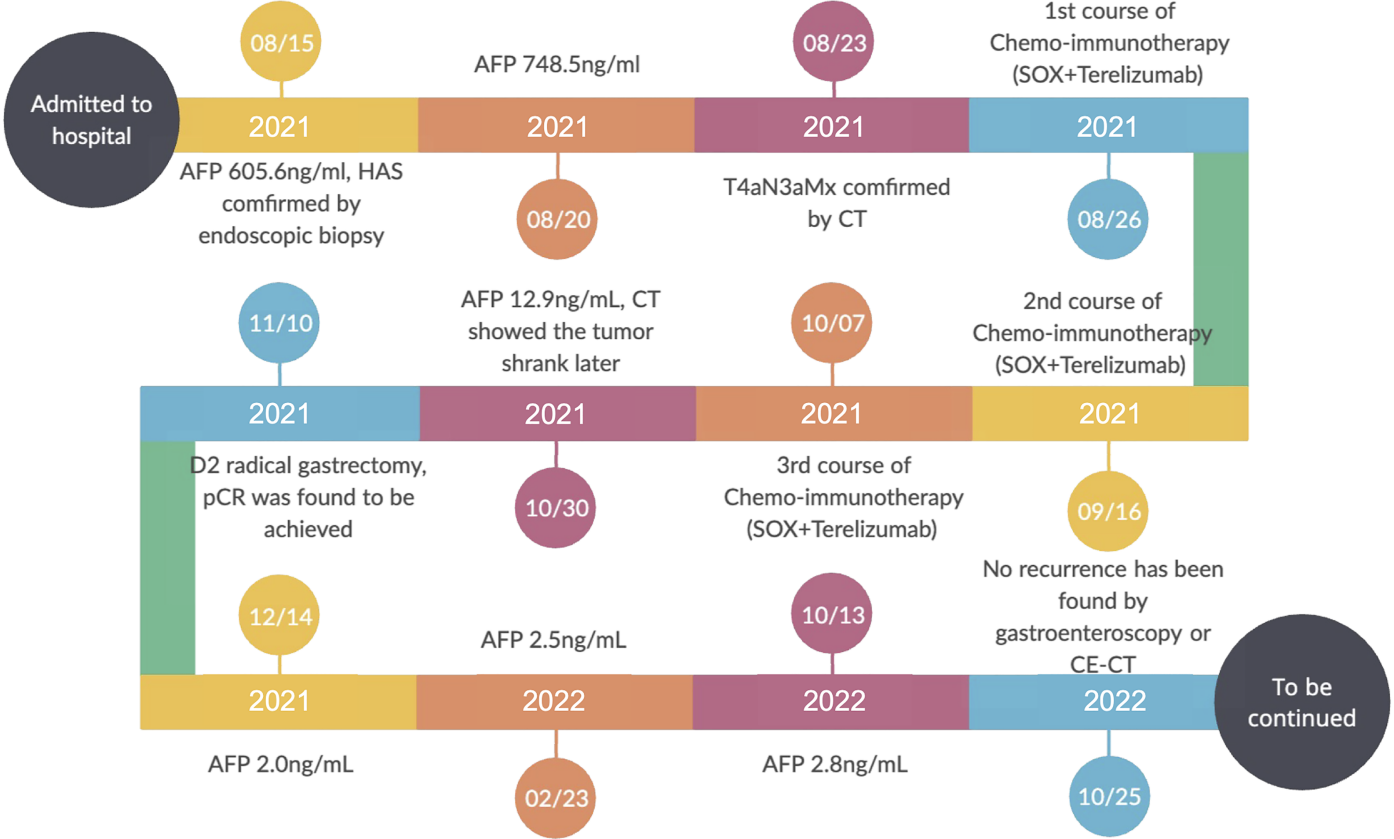
Timeline of the clinical course.

**Figure 2:**
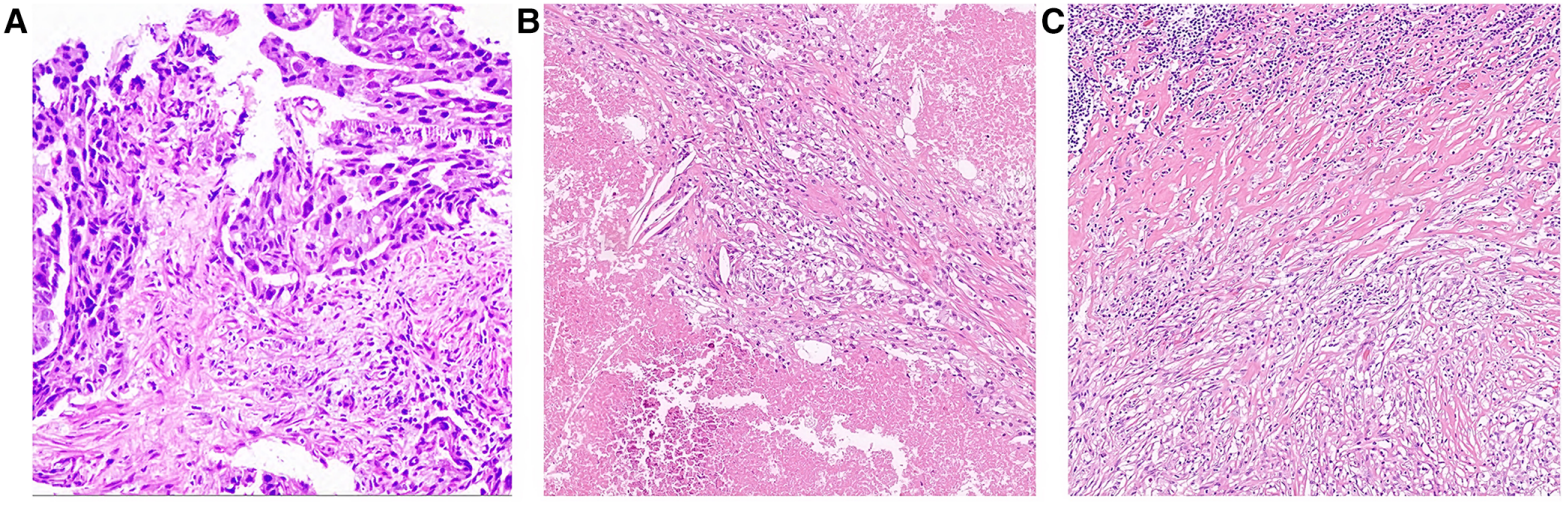
Pathological findings (**A**) Endoscopic biopsy before chemo-immunotherapy at local hospital (Hematoxylin-eosin staining, magnification ×10); (**B**) Tumor area and (**C**) lymph node after 3 courses of chemo-immunotherapy (Hematoxylin-eosin staining, magnification ×10).

**Figure 3:**
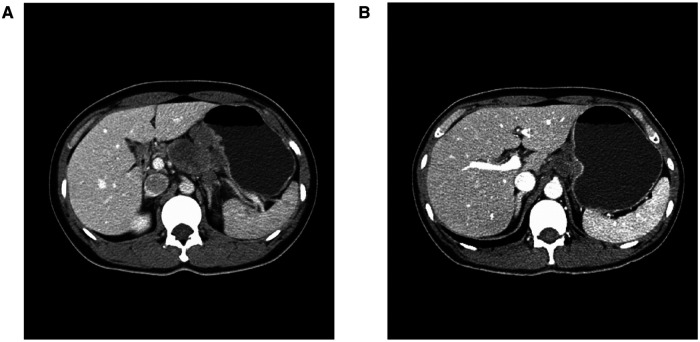
Abdominal CT imaging (**A**) Before chemo-immunotherapy; (**B**) After 3 courses of chemo-immunotherapy.

As shown in the clinical time line of Figure 1, a 48-year-old woman was admitted to the local hospital due to highly elevated serum AFP level during a yearly routine physical examination. She underwent upper endoscopy and was found to have HAS in the cardia verified by pathological examination (Figure 2A). With no notable family history and no history of smoking or drinking, she did not complain of any obvious symptoms. After that, she was then referred to our hospital for further treatment. The results of blood routine, biochemical tests, and coagulation tests were within the normal ranges. Contrast-enhanced CT (CE-CT) scan showed that the wall of the gastric fundus and cardia was thickened and enhanced, and multiple lymph nodes were found with enlargement. The tumor was thus diagnosed as T4aN3aMx and Borrmann type III (Figure 3A). Immunohistochemistry (IHC) showed c-erbB-2(GC) 1+, c-ervB-2(GC)-Positive Control 3+, MSH2+, MSH6+, MLH1+, PMS2+, SALL4+, AFP+, GPC3+, Syn−, CgA−, CD56−, and Ki-67 80%+. Genome sequencing showed microsatellite stable (MSS) in tumor tissues. PD-L1 expression level was also tested using the PD-L1 IHC 22C3 pharmDx. A specimen with combined positive score (CPS) ≥1 is considered to have PD-L1 expression but the patient's tumor had a CPS of <1, thus revealing a negative PD-L1 expression.

However, the high efficiency and low toxicity of ICIs have been confirmed by previous clinical studies (Table 1) and with respect to the patient's wishes, a multidisciplinary decision was made to trial a neoadjuvant therapy regimen including oxaliplatin plus S-1 (SOX) and PD-1 inhibitor terelizumab every 3 weeks (S-1, 100 mg/day, orally on days 1–14; oxaliplatin, 130 mg/m^2^, intravenously on day 1; terelizumab, 200 mg, intravenously on day 1; the patient had a weight of 45 kg and a height of 156 cm). After three courses of the combined therapy, the serum AFP level decreased from 748.5 to 12.9 ng/mL (Figure 1) and the tumor shrunk (Figure 3B). D2 radical gastrectomy was then performed and no residual tumor cells were found in resected tissue; most of the cells appeared to degenerate and die (Figures 2B,C). Hence, the disease might have been considered as a partial response (PR), but the postoperative pathology revealed that a pCR had been achieved. As for the adverse effects during the treatment, the patient presented some common symptoms like fatigue, loss of appetite, and nausea, which however could be tolerated well by adopting symptomatic therapies. To date, the patient in this study has been followed up for more than a year after the radical surgery. AFP level has been around 2.5–2.8 ng/mL during the past 10 months and no evidence of recurrence has been found by gastroenteroscopy or CE-CT.

**Table 1:** The cases of complete response after immunotherapy in combination with/without chemotherapy/targeted therapy.

No	Reference	Gender	Age (years)	Tumor type	Chemotherapy/Targeted therapy	Immunotherapy	PD-L1 expression	Outcome
1	Liu et al. 2022 (10)	Male	74	Gallbladder carcinosarcoma	Gemcitabine** **+** **oxaliplatin (GEMOX)	Sintilimab	+	CR
2	Yi et al. 2022 (14)	Female	74	Gallbladder carcinoma	S-1	Pembrolizumab	**−**	CR
3	Guo et al. 2022 (15)	Male	42	Leiomyosarcoma	Pazopanib, anlotinib	Pembrolizumab	**−**	pCR
4	Locati et al. 2021 (16)	Male	62	Head and neck squamous cell carcinoma	N/A	Nivolumab	+	CR
5	Brochard et al. 2022 (12)	Male	74	Colorectal adenocarcinoma	N/A	Nivolumab, ipilimumab	N/A	pCR
6	Takami et al. 2021 (17)	Female	70	Gastric adenocarcinoma	N/A	Nivolumab	N/A	CR
7	Ansari et al. 2021 (18)	Female	45	Vaginal squamous cell carcinoma	N/A	Pembrolizumab	+	CR
8	Gallois et al. 2022 (19)	Female	58	Colorectal adenocarcinoma	Encorafenib, cetuximab	Pembrolizumab	N/A	pCR
9	Wang et al. 2021 (20)	Male	26	Hereditary leiomyomatosis and renal cell cancer	N/A	Pembrolizumab	+	CR
10	Hu et al. 2021 (21)	Male	61	Non-small cell lung cancer	Paclitaxel, carboplatin	Pembrolizumab	**−**	pCR
11	Liu et al. 2021 (22)	Female	49	Hepatocellular carcinoma	Bevacizumab	Atezolizumab	N/A	CR
12	Sekido et al. 2021 (23)	Male	59	Oral squamous cell carcinoma	N/A	Nivolumab	+	CR
13	Higuchi et al. 2021 (24)	Male	73	Non-small cell lung cancer	N/A	Pembrolizumab	+	pCR
14	Lin et al. 2021 (25)	Male	78	Gastric adenocarcinoma	Capecitabine	Pembrolizumab	+	CR
15	Wang et al. 2021 (11)	Female	66	Bladder urothelial carcinoma	N/A	Sintilimab	N/A	CR
16	Bucalau et al. 2021 (26)	Male	71	Hepatocellular carcinoma	Doxorubicin (TACE)	Nivolumab	N/A	CR
17	Magalhaes et al. 2021 (27)	Female	69	Bladder urothelial carcinoma	N/A	Atezolizumab	N/A	CR
18	Li et al. 2021 (28)	Male	66	Gastroesophageal junction carcinoma	S-1 + oxaliplatin (SOX)	Camrelizumab	**−**	pCR
19	Tang et al. 2021 (29)	Male	60	Pancoast tumor	Pemetrexed, nedaplatin	Tislelizumab	+	pCR
20	Takeuchi et al. 2021 (9)	Male	84	Non-small cell lung cancer	N/A	Pembrolizumab	+	CR
21	Iribe et al. 2021 (30)	Male	49	Hereditary leiomyomatosis and renal cell cancer	N/A	Nivolumab, ipilimumab	+	CR
22	Rao et al. 2020 (31)	Female	70	Gallbladder carcinoma	Apatinib	Camrelizumab	+	CR
23	Matull et al. 2020 (32)	Male	70	Melanoma	N/A	Nivolumab, ipilimumab	**−**	CR
24	Wang et al. 2020 (33)	Male	55	Non-small cell lung cancer	Gemcitabine, cisplatin	Durvalumab	+	pCR
25	Fricke et al. 2020 (34)	Female	66	Non-small cell lung cancer	Carboplatin, pemetrexed	Pembrolizumab	+	CR
26	Zhu et al. 2020 (35)	Male	54	Sarcomatoid hepatocellular carcinoma	N/A	Nivolumab	+	CR
27	Xue et al. 2020 (36)	Male	54	Non-small cell lung cancer	N/A	Toripalimab	+	CR
28	Zhang et al. 2020 (37)	Female	86	Small cell lung cancer	N/A	Pembrolizumab	**−**	CR
29	Tang et al. 2020 (38)	Male	68	Non-small cell lung cancer	N/A	Pembrolizumab	+	pCR
30	Liu et al. 2019 (39)	Male	63	Hepatocellular carcinoma	Lenvatinib	Pembrolizumab	N/A	CR
31	Abdallah et al. 2019 (40)	Male	90	Merkel cell carcinoma	N/A	Avelumab	N/A	pCR

TACE, transarterial chemoembolization; PD-L1, programmed cell death ligand-1; CR, complete response; pCR, pathologic complete response.

## Discussion

Ever since first identified as “AFP-producing gastric cancer” in 1970s (41), HAS has still been underrecognized due to its rarity in clinical practice (42). Although various cases and retrospective reports of small sample size regarding HAS have been reported over the years (3, 43–48), it has not been sufficiently understood, resulting in a high mortality and terrible prognosis. Herein, we presented a rare case of HAS that was successfully treated with combined SOX and terelizumab. To the best of our knowledge, this is the first report of pCR in HAS from combined chemotherapy and anti-PD-1 therapy.

Previous reports have discovered that the high-frequency gene alternations in HAS tumor samples including TP53, RPTOR, CD3EAP, CEBPA, WISP3, and MARK1, among which TP53 was the most frequent one. Apart from gene mutation, HAS is also a malignancy with a remarkable augment of copy number gains (CNGs) (3). In particular, patients with CNGs situated in 20q11.21–13.12 of a chromosome with a trend of increasing serum concentration of AFP have been reported to have worse prognosis (44). Here in this case, although the patient refused to perform further additional molecular analyses, it was speculated that she might be lucky enough to avoid such CNG due to the less aggressive bio-behavior of the tumor.

Given the limited randomized controlled trials (RCTs) regarding the HAS, no consensus on the ideal treatment approach for the disease has been reached. Based on previous published literature, most of the cases received radical surgery with adjuvant chemotherapy (1, 8, 49–53). Despite no recommended standard regimen of chemotherapy, adjuvant chemotherapy has been confirmed as one of the independent factors of long-term survival (7, 54). It has been reported that SOX (49) or FOLFOX (52) might be potentially favorable protocols for HAS. In addition to systemic chemotherapy, interventional therapy with relatively less toxic side effects could be used when a liver metastasis exists and targeted therapy might be an alternative when resistance to chemotherapy occurs (55).

In recent years, anti-PD-1/PD-L1 immunotherapy has attracted great attention and revolutionized the treatment landscape of various solid tumors. The general underlying mechanism of cancer immunotherapy is to stimulate an immunologic reaction to inhibit the tumor immune escape from the immunologic surveillance system (37). However, it has barely been reported in HAS, let alone pCR cases that benefited from immunotherapy. In this study, it has been suggested that neoadjuvant immunotherapy plus chemotherapy followed by surgery might offer an advantageous or alternative method for treating HAS. Further evaluation of ICIs and their combination treatments in the perioperative setting might be beneficial for patients with locally resectable disease (28).

Although immunotherapy is right now in the center of the spotlight of anticancer battlefield, there are still a number of patients who unfortunately failed to benefit from it (56). Usually upregulated in various tumors, PD-L1 expression is the most frequently used biomarker for predicting the potential response to ICIs (57). In this case, what was surprising was that the patient presented with MSS and negative PD-L1 expression who was supposed to be relatively insensitive to immunotherapy and found that she still had an excellent response to anti-PD-1 antibody combined with chemotherapy, indicating that other mechanisms than MSI and PD-L1 positive might account for the responsiveness to ICIs (28). As shown in Table 1, this phenomenon has also been reported in gallbladder carcinoma (14), leiomyosarcoma (15), non-small cell lung cancer (21), gastroesophageal junction carcinoma (28), melanoma (32), and small cell lung cancer (37). Previous studies demonstrated that chemotherapy might be able to enhance anticancer immunity by reactivating immune effector cells, stimulating tumor antigen presentation, and eliminating immune suppressor cells, thus resulting in a synergistic anticancer effect compared with the anti-PD-1 monotherapy (58, 59). Recently, a meta-analysis has also found that the early death rate upon immune checkpoint inhibitors across solid malignancies was not predictable by PD-L1 expression (60). This is an area that requires further exploration in future.

However, given the single-case retrospective nature of this study, the major limitation might be the insufficient evidence to support the benefit of the combined treatment. The understanding of the biological behavior of HAS is still limited due to its rare incidence. Moreover, the mechanisms of the current anti-PD1 therapeutic strategies have not been fully elucidated. Nonetheless, this case report sheds some new light on the treatment of HAS. It is anticipated that both clinical and basic research will continue to advance with the accumulation of future HAS cases.

## Conclusions

To conclude, this study presented a rare case of HAS with negative PD-L1 expression which, however, achieved pCR following chemotherapy (SOX) in combination with immunotherapy (terelizumab), providing a novel perspective on the potential treatment strategies for this aggressive malignancy. Further studies are highly warranted to explore the underlying mechanisms of the combined chemo-immunotherapy and improve our understanding regarding the management of HAS.

## Data Availability

The original contributions presented in the study are included in the article/Supplementary Material, further inquiries can be directed to the corresponding author.
